# What Makes a Good Theory, and How Do We Make a Theory Good?

**DOI:** 10.1007/s42113-023-00193-2

**Published:** 2024-01-24

**Authors:** Olivia Guest

**Affiliations:** 1 Donders Institute for Brain, Cognition, and Behaviour, Radboud University, Nijmegen, Netherlands; 2 School of Artificial Intelligence, Radboud University, Nijmegen, Netherlands

**Keywords:** Theory, Metatheory, Metatheoretical calculcus, Theoretical virtue, Metascience

## Abstract

I present an ontology of criteria for evaluating theory to answer the titular question from the perspective of a scientist practitioner. Set inside a formal account of our adjudication over theories, a *metatheoretical calculus*, this ontology comprises the following: (a) *metaphysical commitment*, the need to highlight what parts of theory are not under investigation, but are assumed, asserted, or essential; (b) *discursive survival*, the ability to be understood by interested non-bad actors, to withstand scrutiny within the intended (sub)field(s), and to negotiate the dialectical landscape thereof; (c) *empirical interface*, the potential to explicate the relationship between theory and observation, i.e., how observations relate to, and affect, theory and vice versa; (d) *minimising harm*, the reckoning with how theory is forged in a fire of historical, if not ongoing, abuses—from past crimes against humanity, to current exploitation, turbocharged or hyped by machine learning, to historical and present internal academic marginalisation. This work hopes to serve as a possible beginning for scientists who want to examine the properties and characteristics of theories, to propose additional virtues and vices, and to engage in further dialogue. Finally, I appeal to practitioners to iterate frequently over such criteria, by building and sharing the metatheoretical calculi used to adjudicate over theories.

## Introduction

Science, too, is a form of art that not only deforms reality but constitutes it as such.

Kofman , 1993, p. 156

Scientists do not conjure theories out of thin air, but as a function of our experiences with the world in general and specifically with our colleagues, through dialogue, and the literature. Our sources of inspiration contain both empirical observations and theoretical positions. Tales of solutions to scientific problems coming to practitioners in their dreams (albeit probably untrue in the case of Kekule and the structure of benzene; Rudofsky and Wotiz , 1988) have merit in that they highlight that often the collection of experiences we have merge into useful wholes without us needing to take any specific formal steps, or so it seems. Even though the process of theory creation might be mysterious or even unknowable in certain cases, theories have a genealogy worthy of examination—if not to infer their exact generation process, then to minimally chart through time the progression of their values, of their ripple effects on the rest of society, of their interface with their fields, and of their internal properties.

The adjudication over theories that we collectively do as scientists by taking into account their theoretical virtues, i.e. their desirable properties, merits examination, and even formalism, into a ‘metatheoretical calculus’ (Guest & Martin, 2023). In so doing, we may explicitly separate a proposed map from the territory of our (meta)theoretical reasoning (Korzybski, 1933). An important step prior to formalisation of such meta-scientific and -theoretical processes is to decide which (types of) potential theoretical qualities or characteristics are worthy of taking into account when modelling this landscape. This step is what this paper is about. There are multitudinous virtues and vices we can evaluate theories against, and being explicit about which we value is part of formal and open (meta)theorising (viz. Guest and Martin , 2021, 2023).

Herein, I explore and unpack what properties, virtues and vices, are both possible and desirable for us as scientists. This includes mixing and matching, as well as articulating our own criteria for our theories. As such, the intended audience of this work is science practitioners, especially those who carry out experiments, who perhaps have not had the opportunity yet to examine the theories they use in non-empirical ways, or who do not engage explicitly with metatheory. Typical framings of such criteria, as presented traditionally in the philosophy of science, deal with the internal components of theories or their relation to evidence; herein, however, virtuous and vicious aspects of theory include its relationships to other theories, its embedding in its field(s) broadly, and in society at large. These typical framings are also often imposed from outside by philosophers. In the following sections, I expound on all such potential candidate criteria, setting them in an ontology and delimiting what scientific objects we treat herein as theories.

## Why a Calculus? Why an Ontology?

Creating one’s own evaluative framework of virtues and vices for theories is what most scientists are already doing, albeit implicitly. Practitioners have their favourite theories, and they choose what to spend their research time on, which theories to criticise, to abandon, and even which they decide to call pseudoscientific (e.g. Fleming et al. , 2023; Spanton and Guest , 2022). All these ebbs and flows of accumulating, (re)building, and abandoning theoretical or empirical work boil down to factors under scientists’ control in many cases, such as what theoretical framework to operate under, which methodology to use, and what assumptions to make. As Guest and Martin (2021, 2023) argue and demonstrate, such processes benefit from formal scrutiny at the theory and metatheory levels, e.g. by sketching out models of scientific practice.

The hitherto un(der)examined decision-making, categorisation, ranking, selection over, between, theories deserves deep thought, discussion, and formal treatment—that I describe as adjudication over theories and propose should be formalised into ‘metatheoretical calculi’ (Guest & Martin, 2023). I argue for a two-fold change in how we, as scientists, relate to our theories.

First, I propose we define, conceptually engineer, and scope ‘theory’, and related concepts. Not into a monolithic or prescriptive unity, but into communicable and useable concepts that each scientist for themselves can create and as a function of their requirements. Such issues are not typically on our minds, if ever, when we discuss theories. A typical evaluation of theories by cognitive or psychological scientists, for example, takes the form of empirical comparisons, e.g. quantitative fit of inferential models of the data or of computational models of the phenomena (e.g. Levering et al. , 2020). Herein, I am interested in including more criteria for properties, especially the un(der)discussed aspects, of theories. To do this, however, I propose we need to carve out what each of us sees as a theory, how theories relate to each other, and their larger embeddings in science and society. This will be unpacked in the section: ‘Metatheory, Theory, and Calculi Thereof’. A formalisable way of looking at theories—that I dub a metatheoretical calculus—is not to automate nor set in stone the ways in which we think through repercussions of our ideas about, e.g. theoretical vices, but to allow for clear reflexive thinking.^1^

Second, I present the idea that we, as scientists, can construct our *own* set of virtues and vices from the full ontology through selection and augmentation—that we can be aware of how metatheoretical maps are drawn of our theorising. We do not need to leave this exclusively to the purview of philosophers of science. We can actively engage with adjudication over this activity that we otherwise just carry out. In the same way, we formally clarify what we mean by theory, and we must do the same for what we mean by *virtuous* theory. This is what I present in ‘Virtue and Vice’, an ontology, summarised in Table 1, which can be seen as synthesising the syntactic, semantic, and pragmatic views on scientific theories (cf. Lutz , 2015; Morrison , 2007; Morgan and Morrison , 1999; van Fraassen , 1977; Cartwright , 2004; Cartwright et al. , 2020). In other words, the traditional theoretical virtues found in the philosophy of science, such as accuracy, consistency, fruitfulness, simplicity, and scope (e.g. Keas , 2018), pertain to the internals of the theory or its relation to evidence. I include these in the ontology presented here, but I also present the possibility of including aspects of theories that involve their relation to other theories, to the field(s) they are embedded in, to the whole of science, and to society in general.

**Table 1:** The proposed ontology with descriptions of what kinds of properties of a theory, i.e. which aspects can be evaluated as virtuous or vicious, is captured in the column labelled ‘Category’

*Category*		*Potential questions*
*a*) **Metaphysical commitment**, the need to highlight what parts of theory are not under investigation, but are assumed, asserted, or essential		What is the theory? What causal relationships does the theory propose? What does the theory assert? In which field(s) is it embedded? What counts as (mis)use?
*b*) **Discursive survival**, the ability to be understood by interested non-bad actors, to withstand scrutiny within the intended (sub)field(s), and to negotiate the dialectical landscape thereof		Is the theory accessible, easy to understand? Are members of the theory’s community able to explain the theory? Are the theory’s concepts transparent? Are labels used consistently in discussions? Can relevant outsiders understand the theory?
*c*) **Empirical interface**, the potential to explicate the relationship between theory and observation, i.e. how observations relate to, and affect, theory and vice versa		What formalisations are appropriate or necessary for the theory? Which methodologies does the theory use to support or instantiate itself? What type of evidence does the theory require?
*d*) **Minimising harm**, the reckoning with how theory is forged in a fire of historical, if not ongoing, abuses—from past crimes against humanity, to current exploitation, turbocharged or hyped by machine learning, to historical and present internal academic marginalisation		Does the theory contain great man theorising? Is the theory exclusionary to certain people or ideas? What is its relationship to previous theories? Does the genealogy of the theory contain harmful ideas? Do ancestral theories remain relevant to the present? What harms have resulted or could result from the use of the theory?

Each category (*a* to *d*) emphasises or selects for different aspects of a theory allowing scientists to change focus from merely, e.g. predictive power or parsimony, to (thinking about) what is appropriate or useful for them. From the perspective of each of these four ways of looking at a theory, scientists can generate their own criteria, think deeply, and iterate over their decisions about what makes a good theory inter alia through asking and answering the examples shown in the column ‘Potential questions’ on the right

**Fig. 1:**
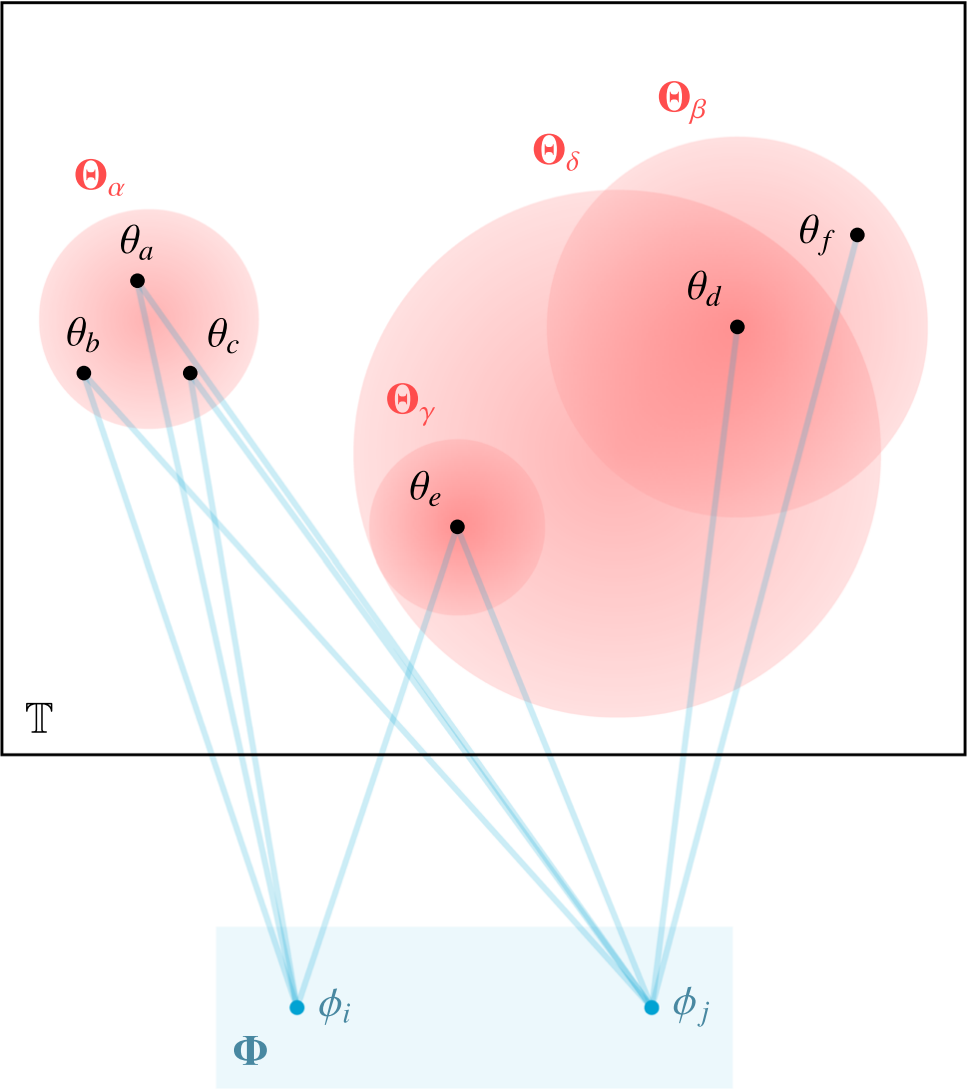
A diagrammatic depiction of theories, $\theta$ (in black), and how they might relate to families of theories, $\boldsymbol{\Theta }$ (in red) within $\mathbb {T}$, which represents a field’s theoretical positions. Any $\theta$ that is part of the field would definitionally need to be placed in (at least one) field $\mathbb {T}$. In this schematic, distance between theories is meaningless, and only family overlap (set intersection) matters, i.e. that $\theta _d$ is in two families: $\boldsymbol{\Theta }_\beta$ and $\boldsymbol{\Theta }_\delta$. Families of theories can intersect, and/or individual theories can belong to more than one family. In cyan are the relationships, the mediation provided by models, between theories $\theta _a$ to $\theta _f$ and the phenomena $\phi _i$ and $\phi _j$. These blueish lines represent that, e.g. $\theta _a$ pertains to, accounts for, both depicted phenomena in $\boldsymbol{\Phi }$

## Metatheory, Theory, and Calculi Thereof

[T]he solution of a great problem often began with the astonishment about a fact which had previously not caused any astonishment and therefore had not been recognized as a problem at all. Charney , 1965, p. III-1

Before we can move to describing what I propose can be done for curating and creating our own virtues and vices in service of our scientific (meta)theorising, I formally organise my thoughts on theory. Such a formalisation is not intended as a prescription. Indeed, it could even be argued that every scientist should create a custom-made calculus to suit their needs for their metatheorising, or at least not adopt the one herein lightly. Creating one’s own formalisations allows taking stock of what is the scientific object that we want to evaluate. What is a theory? What is the remit of our evaluation exercise? How do we constrain the space of where possible virtues may apply? What parts of our science are virtuous or vicious theoretically? What parts of what we do as scientists are we interested in, what counts as theory to us, in what modality is it expressed, and through which lens do we look at it? Once we answer these kinds of questions, we can move on to defining what parts are virtuous, and how such evaluations may be reached. In this section, I offer such answers and ask others to think about their own perspectives.

*Metatheoretical calculus* is an umbrella term for a (semi)-formal system to describe, define, and constrain the process of adjudicating over theories in a given (sub)field. Metatheoretical calculi are for understanding and improving the process of science, for making transparent what definitions and standards for ‘theory’ and related terms are being used by practitioners to enable formal capture of that process (viz. Guest and Martin , 2023). To presage the rest of the paper, such calculi allow for delimiting, deciding on, and communicating the way in which scientists intend theory to be virtuous or vicious.

Metatheoretical calculi do not require one single framework nor formalism, but constitute a proposal that one or more such formal systems might provide useful ways of navigating our metatheoretical ideas. An important consideration that must be addressed before moving to the definitions and formalisms below is the following: It is not the intent of the author to propose that a metatheoretical calculus is a single beast, it can be composed of figures, it can be verbal descriptions, it can be set theoretic, etc. It is the idea that formalising can set us free, allow us to think critically about our own thoughts, in this case about theory, and should not be used to lock us in to a certain way of thinking. Much like formal or computational modelling of phenomena generally, metatheoretical calculi, can be seen as consumable scientific products on the way to deeper insight.

Herein, *theory* is ‘a scientific proposition — described by a collection of natural-language sentences, mathematics, logic, and figures — that introduces causal relations with the aim of describing, explaining, and/or predicting a set of phenomena’ (p. 794 Guest and Martin , 2021).^2^*Phenomenon* is a process that generates, or requires, observations, constituting scientists’ perspective on what things appear or seem to be, not how they are. A phenomenon need not uniquely fit under one theory and indeed can be agreed upon (in terms of existence and/or properties) by contrastive theories, e.g. celestial bodies appear to move in both geocentric and heliocentric theorising.

**Fig. 2:**
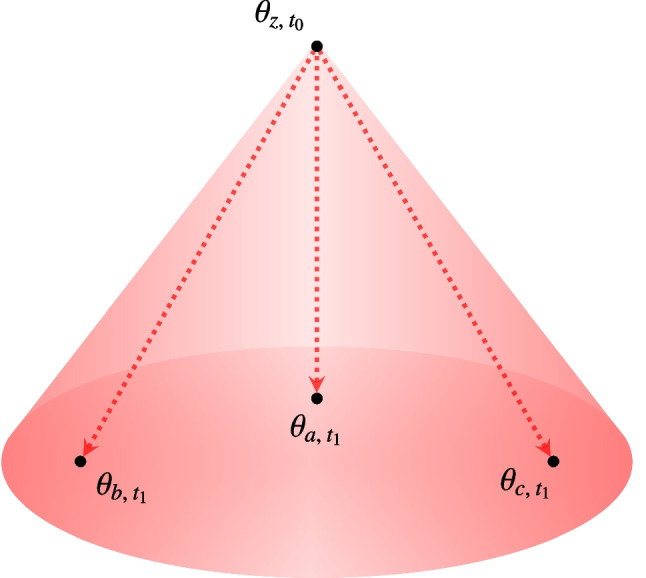
A view of theory family $\boldsymbol{\Theta }_\alpha$ from Fig. 1 seen through time, allowing the practitioner to track genealogical linkage between three related concurrent variants of $\theta$ (at time $t_1$) and their ancestral $\theta _z$ (at a prior time $t_0$, and which can or cannot be an element of $\boldsymbol{\Theta }_\alpha$). The genealogical relationships between $\theta$-theories can be modelled using a directed acyclic graph, for example, and are depicted above using dashed red lines, such that $\theta _i\!\dashrightarrow \!\theta _j$ denotes that *j* descended from *i*. Due to the cartoon nature of this depiction, $\theta$- and $\boldsymbol{\Theta }$-theories have simplistic relationships—a more realistic depiction will likely contain complex linkages within theories and between theories and phenomena (recall Fig. 1)

Figure 1 presents a potential, proof of concept, step towards a formal treatment of the relationships between phenomena, $\phi$, and theories. The latter could be analysed into three possible levels: $\theta$-theories (the smallest unit of a theory), $\boldsymbol{\Theta }$-theories (families of theories), and $\mathbb {T}$-theories (fields). A $\theta$-theory can be the unit of publication, and so families of theories $\boldsymbol{\Theta }$ can contain multiple theories $\theta$, whose relationships can be further detailed using, e.g. citational analysis. A set-theoretic account allows the word ‘theory’ (like ‘set’) to be used for fields and families ($\mathbb {T}$ and $\boldsymbol{\Theta }$, with many elements) as well as individual theories ($\theta$, which may be singleton sets, consisting of a single journal article). Theory, denoted by $\theta$, $\boldsymbol{\Theta }$, and $\mathbb {T}$, can be about any observations or phenomena that we wish to examine under a meta (-scientific or -theoretical) lens. So for example, the conception of a field of study is itself a theory about the field, i.e. what constitutes ‘computational science’ (Birhane & Guest, 2021) or defining the hardness of ‘cognitive science’ (Rich et al., 2021). And so an understanding of a field (which is, the understanding $\mathbb {T}$, itself an instantiation of a theory) can be genealogically and/or thematically linked to other theories (see Fig. 2, which will be discussed more in ‘Virtue and Vice’).

In this particular metatheoretical calculus, theories are related to units of publication (conference talks and posters, journal articles, books, etc.) and not groups of people or individuals. This allows for conceptual clarity on what a given theory comprises, requires, and assumes, e.g. which $\theta$-theories are in which $\boldsymbol{\Theta }$-theories. It also allows for criticism to be clearly delimited as about the work, the scientific output, and not the individual people involved, especially since inter alia authors may have moved on from espousing the published ideas. As with all carvings of the (meta)theoretical landscape I present here, this is a suggestion. It is possible that in some fields, taking a single paper as an instance of a theory might not be useful or sustainable. For example, an individual’s or group’s output could be framed at the $\boldsymbol{\Theta }$- or $\theta$-levels if diverging from the literature is necessary. However, unless interviews are carried out to find out who is in the group, what they believe, what other resources they recruit, at a given time for a given theory, etc., it seems harder to pin this down than to extract these details from the written word, which is also often the main interface to a theory other practitioners will have.

In this piece, the word ‘theory’ has these broad, although still delimited, set-theoretic senses chosen with the aim of proposing an evaluative framework for these types of scientific object. In essence, theory is not discussed herein as a single thing—it is suggested to be organisable, conceptualisable, and contextualisable into $\theta$, $\boldsymbol{\Theta }$, and $\mathbb {T}$. The idea of metatheoretical calculi is a request to scientists to formalise their views on what theory is and how they reason about theory, with the intent of both problematising and exploring their own understanding and of communicating their ways of carving literature, theories, fields, and so on, to others. Explicitly, the notion of an ontology—unpacked in the next section—for theoretical properties is meant to initiate and inspire thought and dialogue, making us mindful of which judgements we make and how we formulate them, forcing us to be explicit via metatheoretical calculi (Guest & Martin, 2021, 2023). Such a formal description of how we think (meta)theoretically allows us to be transparent and consistent, i.e. if some practitioners value falsifiability, or parsimony (cf. Longino , 1996; Harding , 1975), they are invited to think deeply if they apply these criteria fairly and consistently across the board. Relatedly, more criteria and refinements can be proposed in the future, both under the given headings and extending the framework’s ontology, including in the direction of further formalisation or restructuring or overhauling of the formalisms used in the metatheoretical calculus. In the next few sections, we will radically reimagine an ontology of such theoretical properties, setting them inside appropriate metatheoretical calculi.

## Virtue and Vice

There’s many a slip twixt cup and lip.

English proverb (Speake, 2015)

In the proposed ontology, summarised in Table 1—broad overlapping categories or themes—of theoretical characteristics, the absence of a virtue need not necessarily be a vice and the inverse. However, the importance of displaying a virtue or avoiding a vice can be of higher or lower importance as a function of the practitioner’s preferences. Additionally, it is strongly suggested that practitioners generate their own metatheoretical calculi and evaluate theories as scientific objects construed as broadly as possible, like I have done herein. As such, we may transcend traditional views on evaluating theory structure, e.g. allowing for non-, pre-formal theories to be virtuous (also see Morrison , 2007; Morgan and Morrison , 1999).

Herein, the classes of potential or proposed theoretical virtues and vices allow us to heuristically nullify the goodness of a theory in its current state if it appears vicious for those classes. Theories that exhibit vice can be argued to be bad and can be rejected in their current state without detailed appeals to any potential virtues, if the science practitioner so wishes. That is, if a theory can be argued to have a vice, we can move on to examining a better theory and/or work hard towards making it virtuous on that dimension. If a $\theta$-theory lends itself to certain vicious uses or is embedded in a vicious field, that is taken to be a property (to be removed, or otherwise addressed, if possible) of the theory itself. All these nuances are suggested to be placed inside a metatheoretical calculus, i.e. to relate their theoretical adjudication to formal treatments of theory. If $\theta$ has a certain property, then $\theta$ is vicious. If $\theta _{z, t}$ is vicious, then all descendents at $\tau \ge t$ are too (recall Fig. 2).

As will be unpacked, each scientist is free to, invited to, construct their own properties for theories in pragmatic terms, i.e. with respect to the field- and society-embedding. Scientists need not just evaluate their theories on syntactic or semantic grounds, which are internal properties. In other words, I present considering criteria for theory pragmatics that scientists may want to construct and consult—and in many cases may do so before addressing any of a theory’s internals, such as its empirical virtues. To draw an analogy, more or less dangerous vehicles exist, regardless of drivers’ abilities—crashes happen both as a function of human and engineering errors, but in all cases, the safety of a vehicle in terms of its design is pivotal for the driver, passengers, people outside the vehicle, etc. The same goes for theories as scientific, and furthermore conceptual, vehicles: when we design theories, we should ideally strive to make them straightforwardly virtuously usable. To further the analogy, the same goes for the infrastructure we build: travelling in these vehicles is also safer and more useful, as a function of the quality of the network both in terms of its upkeep and its design. A train is unusable without train tracks, a network of stations to call at, maintenance, and so on.

### Metaphysical Commitment

The individuation of, relation to, and embedding of a theory in its scientific ecosystem can be formulated as part of the metaphysical commitment it has made. In other words, through answering questions such as the following, we can tease apart what a given theory commits to: What are the $\mathbb {T}$, $\boldsymbol{\Theta }$, and $\theta$ for a given theory of interest, and what are they composed of? How does our theory react and relate to other relevant, competing, complementary theories? Does the theory persist through time or does every $\theta _i$ dramatically change or even annul previous properties or contradict previous statements, and if so, how? For more, see the first row of Table 1,^3^.

#### Constitution, Identity, Persistence

A good theory may have some way of identifying itself to us and dissociating itself from the rest of the literature, thus facilitating talking about it and contrasting it with other accounts. As such, we may explore the separability of a theory from the rest of the body of work of the authors, field, etc. Relatedly, we may ask if a given theory $\theta _{a}$ at time $t_0$ (denoted by $\theta _{a, \,t_0}$) grants us the ability to delimit it from a newer version of itself (say, $\theta _{a, \,t_1}$), both of which could be elements of family $\boldsymbol{\Theta }$ (see Fig. 2). A $\boldsymbol{\Theta }$-theory can contain many, sometimes only superficially, similar $\theta$-theories (recall Fig. 1). One way to enforce this is to take each individual publication pertaining to the named theory as a separate $\theta _i$. In other words, when we want to be formal about what theory is under our metaphorical microscope, we may benefit from fragmenting family $\boldsymbol{\Theta }_j$ contained in multiple journal articles, and so on, into $\theta _i \in \boldsymbol{\Theta }_j$ for each scientific output *i* that (re)describes the properties of the set of theories housed in $\boldsymbol{\Theta }_j$, as shown in Fig. 1. We could further refine this formal treatment within, say, a journal article by teasing apart how and why the theory can be analysed (e.g. internally or procedurally into theory-building steps,Guest and Martin , 2021; van Rooij and Baggio , 2021). What is and is not in $\boldsymbol{\Theta }_j$ can be decided using some explicit classification, e.g. based on the use of the same theory name or the involvement of the same authors within a set of scientific outputs or on a case-by-case basis. Different theories may need different strategies; in Fig. 2, I used directed acyclic graphs to capture such relationships.

Relatedly, a good theory could be one that delimits which beliefs it commits to or depends on that are not under investigation nor subject to further analysis. For instance, a theory of human memory will likely not entertain the idea that memory might not exist. On the contrary, it might explicitly commit to memory as a metaphysical fact (likely by defining it as comprising a class of phenomena, systems, mechanisms, observations, and so on). The theory provides an account of some aspects of human memory without allowing for questioning of the existence of such a capacity. Such questioning, especially if backed by rhetorical and empirical force, would likely affect the theory in deep ways. This can include the threat of abandonment, but minimally, the theory may require changes to accommodate this attack on its founding principles. Importantly, if changes of any kind occur, theory $\theta _{a,\,t_0}$ becomes $\theta _{a,\,t_1}$ (or could fracture further; see Fig. 2). Theory $\theta _{a,\,t_0}$ can be considered falsified if such language aids the reader, but might not seem to be so since $\boldsymbol{\Theta }$ and/or $\mathbb {T}$ still contain (or permit) $\theta _{a,\,t_0}$-like theories, namely $\theta _{a,\,t_1}$. Notwithstanding, a theory that does not, or cannot, react at all to such events is likely vicious. The same goes for a theory that is so vaguely transmitted or indeed purposefully obfuscated, as to allow confusion between $\theta _{a,\,t_0}$ and $\theta _{a,\,t_1}$.

Other components of a theory’s constitution are the baggage a theory may carry (both implicitly and explicitly as part of its constitution or definition) with respect to causation, function, and mechanism (cf. Cartwright , 2004; Egan , 2017; Darden , 2001; Millikan , 1989; Cartwright et al. , 2020). What commitments is the theory making with respect to causal, functional, and mechanistic assumptions or claims—and what types of causation, function, and mechanism are within its purview? If a practitioner needs a theory that provides a mechanistic account, does a given $\theta$ satisfy these needs? Or simpler still, are the causal relationships between components sensible given what the practitioner believes about the target system?

**Fig. 3:**
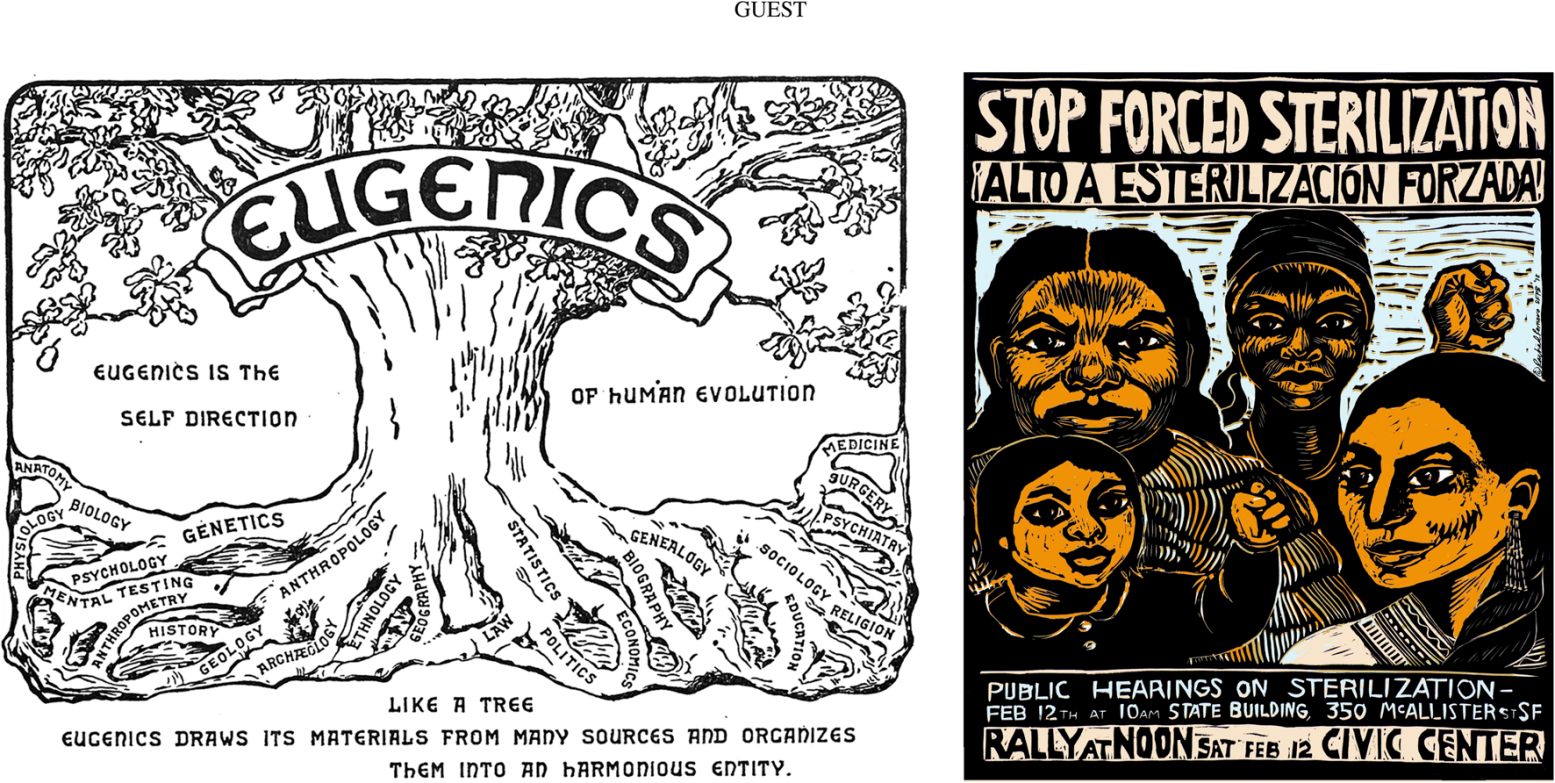
On the left, an example of a theory, eugenics, that not only positions itself as a theory for how to perform and apply human genetics and human biology broadly, but also proposes (to enact) a genealogical relationship between itself and many other disciplines (Cain, 2022). On the right, an example of what is needed to stop the effects of the scientific programme of eugenics. Unchecked by any serious ethical oversight, eugenics became policy (Nourse, 2016). Activism such as that captured by this poster, by Rachael Romero and San Francisco Poster Brigade (1977), was and is pivotal in curbing crimes against humanity, that this theory not only facilitates through granting white supremacist sterilisation programmes scientific prestige, but also eugenics stipulates such abuse is required for the so-called health of our species (Forced sterilizations , 2017; also see Ipgrave et al. 2022). The exact opposite is the case: eugenics around the world needs to end, if we are to preserve our dignity and if we are to ever have a chance of claiming science is a force for good

#### Embedding, Reach, (Mis)use

Also worthy of examination is the flip side of separating the theory from the rest of the field(s): explicitly placing it within the appropriate landscape (cf. Douglas , 2018). Elucidating, in other words, the (clarity of the) field embedding, to what extent is it explicit where in the sciences this theory applies? For example, the answers to questions such as follows: is the family of theories that pertain to the evolution of organisms also applicable outside genetics and biology? A virtuous theory in such dimensions could be required to have a straightforward answer here, not only to avoid misuse and misunderstanding, but also to enhance communication of such novel uses if such a theory were to be adapted or used as inspiration.

In the case of the theory of evolution, there is ‘disagreement between evolutionary theorists and evolutionary psychologists over the proper account of evolution[,] tension between evolutionary psychology and cultural evolution[, and] philosophers of science will doubtless continue to check the credentials of evolutionary ideas imported into other areas of philosophy’ (Downes, 2021). Clearly, practitioners and theoreticians have strong views with respect to the reach and appropriate use of evolutionary theory, including staying up-to-date with theory in evolutionary biology, the purported font of their inspiration, and of their claims to scientific credibility (cf. Ah-King and Nylin , 2010; Roughgarden , 2004). An individual practitioner, a user of such theories, would potentially have to answer such questions for themselves about how they substantiate their use of evolutionary theory to avoid spillover into inappropriate (for them) or even illegal aspects (e.g. crimes against humanity; see Fig. 3 and Burke and Castaneda , 2007). What are the $\mathbb {T}$, $\boldsymbol{\Theta }$, $\theta$, and the relationships between them and the relevant phenomena, which practitioners are comfortable with and which are inadmissible?

Theoreticians exist that could be seen as doing a good job of attempting to buttress their theory and related entities against misuse, e.g. in the case of information theory:

attracted by the fanfare and by the new avenues opened to scientific analysis, [practitioners] are using these ideas in their own problems. Applications are being made to biology, psychology, linguistics, fundamental physics, economics, the theory of organization, and many others. [However,] the establishing of such applications is not a trivial matter of translating words to a new domain, but rather the slow tedious process of hypothesis [testing.]

Shannon , 1956, p. 3

By the same token, there are theories which attempt violently or minimally without the consent of their proponents, to unify competing or alternative accounts all under their own banner, in our calculus likely a $\boldsymbol{\Theta }$ or $\mathbb {T}$ (cf. Andrews , 2021; Bruineberg et al. , 2020). This is not the case with all unification, such as classical mechanics, electromagnetism, and spacetime (Morrison, 2013), which can easily be framed as virtuous. On the contrary, these violent attempts of unification often involve draining meaning from the concepts of alternative theories and replacing them with their own or discarding them completely. Often, the theories that have this property survive and thrive in the mainstream because they are Turing-complete^4^ or otherwise very flexible and/or vague. Theories that act in amoeba-like anti-pluralist ways, encircling, engulfing, and perhaps even digesting other accounts, thus enforcing unification where none is needed nor asked for, likely are vicious and could even be said to damage their fields (also see Longino , 2006; Dow , 2012; Sawyer , 2021; Hibbert , 2016).

### Discursive Survival

Theories are put under investigatory pressure by both proponents and detractors, with likely overlapping aims and reciprocal dialogical strategies (see the second row of Table 1). Metatheoretical calculi can express whether and how a theory can survive such scrutiny as a function of scholarly dialogue. For example, do interested non-bad actors understand the theory as presented by its proponents? Or is reading the collection of works that comprise it somehow insufficient and further non-canonical materials needed even by fellow experts? The answers to such questions are often well known by the community of scholars who engage with the theory, but often the opaqueness and even purposeful obfuscation are seen as a pro not a con. And so, clarity of writing, clear formalisms, and easy-to-understand articles by the proponents of a theory could be framed as a virtue of the theory. If a theory appears to be lacking conceptual transparency to the community as a whole except the proponents, this could be taken to be a sign of weakness (also see Longino , 1996). At a minimum, these issues could be discussed as a clearly admitted cause of concern and area of current improvement.

A core part of good discourse, or of facilitation thereof, can be the transparent use of words and concepts. Especially if they are used by the broader field, or even science in general, in differing ways, it could be made very clear they are being used in specific theory-, or framework-, bound ways. In the case of words, an explicit intervention for disentangling semantics is not perhaps needed for ‘memory’ pertaining to human cognition and ‘memory’ pertaining to digital circuits because the brunt of this disentangling work is carried by the contextual cues, e.g. the field embedding, of the theory. Notwithstanding, there are indeed cases where confusion emerges exactly because of the potential for this type of sloppiness, such as in the field of cognitive computational neuroscience, where words like ‘neuron’ pertain to the map (the smallest computational unit in artificial neural networks) and the territory (a type of cell in the neural system) in ways that can be so unclear as to cause a theoretically damaging merger of the two possible notions (Guest & Martin, 2023).

Another example where effort is needed to tease apart in readers’ minds lexical and semantic differences can be seen in ‘simulation’ as used by computational scientists broadly and the notion embodied cognition theorists imply when they use it. In the first case, simulations typically involve some aspect of running, or an instance of a computational model, e.g. a model with a given set of parameters fixed to certain values. ‘Simulation’ is also sometimes deployed where there is a lack of, or no need for, theoretical understanding in order to instantiate the system, e.g. a flight simulator is not created to further theoretical work but to train pilots, and a weather model can be described as a simulation because it does not embody theoretical instantiation or exploration of the climate, but rather a series of (nonetheless extremely valuable) predictions buoyed by real-time data constantly fed into the model. On the other hand, a proponent of the embodied cognition thesis may use it as an alternative (but not a synonym) to the role mental representations play (e.g. Pecher and Zwaan , 2005). Because this second use case is irregular, or at least in the minority, there is some pressure on the users to carefully and clearly explain their use of the word, which may be familiar as a lexical item to their readers (from daily life and/or from being computationally trained). Coherent explanations in such cases can be seen as adding to the virtuousness of our theories, since we fortify them against misunderstanding.

In terms of concepts with varying labels, similar care could be taken to make seemingly similar entities indeed sensible given divergent embeddings, especially if shared or borrowed from other fields. For example, the concept of biological plausibility makes sense in biology, but does not seem to stand up to further scrutiny in many situations, and in fact reduces to the absurd when discussing neurocognitive modelling at any level above that of biological neurons (Guest & Martin, 2023). A more appropriate concept, constraint, to make theories within the cognitive sciences relate to explananda and/or fall within certain classes of potential accounts is, e.g. computational plausibility (van Rooij, 2008). Another potential solution could be to tether biological or other types of plausibility very carefully to formalism (van Rooij & Baggio, 2021).

Similar hashings out of conceptual nuances, which may seem minor but are pivotal for the discursive health of a theory, are seen in the changing definitions, as a function of refinement, of clinical patient groups (e.g. Neary et al. , 1998; Barker et al. , 2022), in the discussion over the (mis)use of Markov blankets (e.g. Bruineberg et al. , 2020), and in proposed ontology, into weak and strong, of emergent properties in complex systems (e.g. Bersini , 2012). These all constitute examples of ongoing theoretical work, likely earnest attempts to enhance transparency—and could be accommodated under the heading of conceptual engineering (cf. Chalmers , 2020; Kitsik , 2022; Belleri , 2021; Sawyer , 2021).

### Empirical Interface

Aspects such as model fit requirements, how hypotheses relate to data, and what statistical tests are appropriate and what their results mean for the theory can be thought of as providing the empirical interface of theory, whether $\theta$, $\boldsymbol{\Theta }$, or $\mathbb {T}$. If a theory, say $\theta _{z,\,t_0}$ (a theory at a specific time point; see Fig. 2) seems untestable or unfalsifiable that would characterise the lack of (clarity on) interfaces with the observable world, guiding us to perhaps rectify this and create $\theta _{a,\,t_1}$. Often, however, the relationship between a theory (of any type; recall Fig. 1) and observation or evidence is not related coherently or explicitly to phenomena in $\boldsymbol{\Phi }$, and such an oversight could be framed as vicious if these relationships are central to a practitioner’s science (cf. Adolfi et al. , 2023). These relationships are represented by the lines in Fig. 1 that connect $\theta$-theories to phenomena, attempting to capture which $\phi$ are relevant for a given $\theta$—this is summarised in the third row of Table 1.

#### Formalisation

While not all otherwise virtuous theories in their current state are ready to interface with observations, nor generate hypotheses, and so on—and this need not detract from their current virtue—there should ideally be an eye towards such interfacing. As such, clarity is invaluable (recall the relationships between theories and phenomena depicted in Fig. 1), even if the present instantiation of a theory states it is premature to relate to, e.g. raw data (assuming this is a stated goal by practitioners). Interfacing can be done by the mediation provided by formal models, where a given theory generates, or permits, various modelling accounts consistent with its core claims, which in turn allow curation and association with observations and allow these interpreted findings to inform, reform, and refine the theory (Morgan & Morrison, 1999; Guest & Martin, 2021).

To help us understand this part of theory development, theories could be describable as pre- or post-formal. A given $\theta _i$ can be presented by proponents either in a pre- or a post-formalised state, and obfuscation or confusion, e.g. pseudo-formalism, can ideally be avoided (by proponents) and condemned (by practitioners generally). Whether formalism has been carried out yet should not be an open question for a given instance within a $\boldsymbol{\Theta }$-theory—and vice versa, formalism without clear delimiting of the pragmatics of the terms and the intended application is minimally errant and maximally vicious. One could go further and say that $\boldsymbol{\Theta }$- and even $\mathbb {T}$-theory, so whole families and fields could request from their constituent $\theta$-theories (i.e. from their proponents) to state if they are in a pre- or post-formalised state. Obfuscation with respect to the current state of the theory’s substance, pre- or post-formal, could be conceived of as vicious. Importantly, however, being formal (or having formalised or furthermore computational components) while indeed adding buoyancy to a theory can nonetheless leave the door open to theoretical vice, e.g. if the formal components are murkily defined or used to dazzle the rest of the scientific community (also see Guest and Martin , 2021).

#### Methodology

Detailing explicitly which types of research methods and methodologies are relevant to a given theory is often left implicit (an appealing example of a mapping of the methodology space can be found in Håkansson , 2013). Notwithstanding, such constraining of the space of possible methodological paths is imperative to avoid many issues on the level of data collection and inferential statistics, if and when applicable. The results of such inferential statistical and empirical work broadly construed should also be held to affect the theory in predictable ways. In other words, if a theory, e.g. via its mediating formal and/or computational models, permits a variety of tests, and the tests return a certain pattern of results, these interpreted observations should have clearly expected effects of strengthening, weakening, or changing the theory.

### Minimising Harm

Investigating harm, to the field itself as well as to the wider world, is often not an explicitly stated goal for theorists. Examples of this harm are, from cognitive and related sciences, e.g. promoting dehumanising and contorted self-images, which have only recently been discussed by practitioners themselves from the inside of these disciplines (viz. Birhane and Guest , 2021; van Rooij et al. , 2023; Spanton and Guest , 2022; Forbes et al. , 2022; van der Gun and Guest , 2023; Erscoi et al. , 2023; Barlas et al. , 2021). Such an emphasis is not only scientifically advantageous, but also an ethically unavoidable facet of our work. As such, we explore how to delimit and disentangle ethical considerations without futurology, by evaluating past and present; see fourth row of Table 1. At the extreme end of the spectrum, work such as that done in artificial intelligence (AI) with monstrously large artificial neural networks pollutes the atmosphere with climate-changing emissions at unprecedented scales (Strubell et al., 2019) for little benefit and arguably much harm (e.g. Bender et al. , 2021). Such work, for example, contributes directly to making our planet, which ultimately houses science, less habitable (also see Crist , 2013; Brevini , 2020; Inoue , 2018; Thierry et al. , 2023).

#### Great Man Theorising

Most of the work on women scientists fits the “history of great men” mold, with women simply substituted for men. Thus, we have many biographies of great women scientists. These biographical studies of women scientists, for the most part, place the achievements of Marie Curie or Rosalind Franklin within the male world, demonstrating that women have, in fact, made important contributions to what has been defined as mainstream science. Yet the focus remains on the woman as exceptional—the woman who defied convention to claim a prominent position in an essentially male world. One of the problems with this approach to history is that it retains the male norm as the measure of excellence.

Londa Schiebinger, 1987, p. 314

On the one hand, it is common knowledge that scholarly activities are the fruit of the labour of teams of so-called great men’s students, mentees, collaborators, and partners. While on the other hand, the formal archival record gives a dramatically different account attributing such scientific outputs solely to individual men, and more recently individual people generally. To wit, great man theorising is ‘an important obstacle to building theory’ (van Rooij , 2021; also see van Rooij , 2022). Great man theorists fabricate and/or obscure anecdotal and historical evidence to ensure the credit is directed at the target prestigious figure. For example, Pythagoras is overwhelmingly named and credited, but he had many women disciples, who were philosophers and mathematicians in their own right, whose writings survive to this day, and after his death, Theano (a woman, possibly his wife) took over as head of his academy (Schiebinger, 1987; Wider, 1986). To give another example, Lavoisier, the chemist credited with discovering oxygen, was also a woman: Marie Anne Paulze Lavoisier. She could have been credited jointly with her husband, but was not (Eagle & Sloan, 1998).

Further, still, the field of psychology is guilty of degrading the record of past discoveries, of reifying and elevating great men through metatheoretical commitments to their existence, even when they are not in the same nor a neighbouring field. Kekule not only likely confabulated the story of the dream, but also he was not the first chemist to solve the problem of benzene (Rudofsky & Wotiz, 1988). Amusingly perhaps, this weaves together the propensity of scholars to create so-called great men, to not only prop up their theories of the subconscious mind, but also of how science is carried out.

Great man theorising requires the (re)orientation of the theory to direct all credit to one person (or a biased subset of a select few) reminiscent of monarchy—far from the pluralistic or meritocratic facade science often hides behind—where few are in power and get credit, while many toil unseen (see Birhane and Guest , 2021, for more on how to deal with this). In reality, none of the research would be possible without all the laboratory members and students. Even if (in the unlikely case) the singular so-called great person is indeed generating all the ideas, the mental space they enjoy is only possible by the grace of their students, mentees, and employees.

A field $\mathbb {T}$, or constituent $\boldsymbol{\Theta }$ and $\theta$, could be displaying great man theorising if a very small group of peoples’ names appear in literature analyses of acknowledgements, author lists, etc. The metatheoretical account here would need to explain why such a small group of people are cited by so many papers or why they are being described by historical accounts of the field as so-called founding fathers or equivalent, if not to provide a substrate for great men theorising.

#### Exclusionism

Relatedly, the exclusion of ideas or people from certain fields or theory ecosystems can also be framed as bringing about viciousness. To give some examples, within a scholarly discussion on how to understand, theorise about, open science, ideas about capitalism, feminism, equity, diversity, and inclusivity are (or have been) often purposefully pushed aside or explicitly not allowed within mainstream conversations (e.g. Whitaker and Guest , 2020; Mirowski , 2018; Shiffrin et al. , 2021). Cognitive science largely rejected (or was rejected by) anthropology (Bender et al., 2010). And, coloniality, capitalism, and patriarchy deform and stunt theoretical development in science by actively or passively excluding, minoritising, and erasing groups of researchers, such as women of colour or indigenous peoples and their ways of knowing (Prescod-Weinstein (2020); Birhane and Guest (2021); Inoue (2018); Forbes et al. (2022); Larivière et al. (2013); and so on). In other words, there are properties of $\theta$-, $\boldsymbol{\Theta }$-, or $\mathbb {T}$-theories that cause the direct or indirect, purposeful or inadvertent, exclusion of groups of people. Therefore, we could analyse theories as a function of such patterns of exclusion.

Exclusion of ideas and people is a dynamic in which a theory can be embroiled that is different but related to great man theorising—both types of vice are indubitably implicated in both reinforcing each other and socially unjust forces such as sexism and racism. For example, from $\mathbb {T}$-level analysis of AI, we can clearly see theories being erased and/or contributions of the originator being plagiarised through citational exclusion at minimum:

The earliest [AI] researchers were not all men. Margaret Masterman[’s] efforts were contemporary with those of Allen Newell and Herbert Simon, who are generally credited with creating the first working AI program. [...] Until someone writes a seriously revised history of AI, correcting in some ways my own Machines Who Think, Masterman won’t get the credit she deserves.

Pamela McCorduck, 2019, pps. 45–46

Margaret Masterman was out of her time by some twenty years: many of her beliefs and proposals for language processing by computer have now become part of the common stock of ideas in the [AI] and machine translation (MT) fields. She was never able to lay adequate claim to them because they were unacceptable when she published them, and so when they were written up later by her students or independently “discovered” by others, there was no trace back to her[.]

Yorick Wilks, 2000, p. 279

In this example, it is clear how Masterman could have been formulated (instead) as an individual great person, a different vice mentioned above. Instead, she was excluded from the historical record and from citation linkage to her own ideas by virtue of inter alia being a woman, perhaps tempered mildly due to links to extremely academically powerful mentors:

Masterman was one of the first people in the world to attempt machine translation, and she made semantics, not syntax, the driving force. She was deeply influenced by certain aspects of Ludwig Wittgenstein’s later philosophy of language. Despite her gender—Wittgenstein was notorious for his mysogyny—she’d been one of his favourite students[.]

Margaret Boden, 2006, p. xlii

This is even more pronounced in the cases of women who are Black and/or of colour, for example, in AI and computer science, who face barriers in joining such fields ($\mathbb {T}$-theories), whose histories are purposefully obscured, and who are harmed disproportionately by many of the technologies developed (Shetterly, 2016; Benjamin, 2019; Taylor et al., 2021; Kendall, 2011; Atanasoski & Vora, 2019; Koenecke et al., 2020). Ultimately, such people are exploited for their labour and their cognitive capacities (e.g. of collecting and collating data, of scientific knowing, Fricker , 2007). And instead of recognition, they are wronged minimally through citational exclusion and maximally through verbal, physical, and sexual abuse (Chatterjee & Werner, 2021; Budrikis, 2020; Nielsen & Andersen, 2021; Clancy et al., 2014; Hardy, 2014).

#### Geneological Analysis

A radical way to capture and characterise virtue and vice in terms of minimising harm is to analyse the origins and retrace the development of a theory. Theories, $\theta$ and $\boldsymbol{\Theta }$, for example, within psychology have certain genealogical links both to each other and to conceptions, i.e. theories, of the definition of the field itself, $\mathbb {T}$.

A behaviourist framework is genealogically linked to, for example, reinforcement learning and a specific take on the field of (comparative) psychology, inter alia. It is also related to the perspective on human (and other) cognitions granted by the Turing test (cf. Guest and Martin , 2023; Erscoi et al. , 2023). In many ways, a metatheoretical calculus for reinforcement learning as used in AI (e.g. Sutton et al. , 1998), if it took into account genealogy, would have to relate all these elements, such as answering when, how, and why behaviourism is seen as unethical (e.g. applied to treatment or education, Kirkham , 2017; Kumar and Kumar , 2019), seen as not a useful framing of what is attempted to be scientifically understood (Guest & Martin, 2023), or seen as both (Erscoi et al., 2023). These parts of analysing a modern AI reinforcement learning paradigm only drop into place when seen through a genealogical lens.

Another example of genealogical relationships is that between alchemy and chemistry: where the former can be framed as ‘aiming at the production of gold and/or other perfect substances from baser ones, or of the elixir that prolongs life, or even of life itself’ (Pereira, 1998), while the latter could be described as ‘deal[ing] with the properties, composition, and structure of substances (defined as elements and compounds), the transformations they undergo, and the energy that is released or absorbed during these processes’ (Usselman & Rocke, 2023). These two fields are apparently dramatically different, and yet we metatheoretically link alchemy to chemistry in a substantive and canonical way (cf. Chang , 2012)—despite them not both involving turning base metals into gold—and we chose not to link alchemy (also) to say biology, which involves the study of life, or medicine which involves prolonging life, which alchemy was involved in studying.

Using the formalism pattern of Fig. 2, which includes a directed acyclic graph, we can relate the set of theories in alchemy to those of chemistry as a whole $\mathbb {T}_{\textrm{alchemy}}\!\dashrightarrow \!\mathbb {T}_{\textrm{chemistry}}$ for the start of that metatheoretical calculus, and we would likely need to explore what purpose severing, simplifying, or mischaracterising the ties between the ancient and the modern field serves. For example, detailing (not just stating) the genealogical link can help highlight Islamophobic, Sinophobic, or otherwise racist ends in the Western conception of this name change, even if not purposeful. On the one hand, ‘Abu Musa Jabir Ibn Hayyan Al-Azdi [721–815 CE] is considered the father of Arab chemistry and one of the founders of modern pharmacy. [...] Jabir is credited with the introduction of experimental methodology into alchemy and the invention of several chemical processes used in modern chemistry’ (p. 52 Amr and Tbakhi , 2007). While on the other hand, the mainstream retelling of the transition $\mathbb {T}_{\textrm{alchemy}}\!\dashrightarrow \!\mathbb {T}_{\textrm{chemistry}}$ is usually set firmly in the chemical revolution of the eighteenth century, featuring Antoine Lavoisier, the so-called father of chemistry (cf. Eagle and Sloan , 1998). The marginalisation of Jabir could be accomplished by framing him as an alchemist, a pseudoscientist, and/or not a true chemist. The same goes for gunpowder, invented in China in 9 CE by people typically described as alchemists (Needham , 1974; Gwei-Djen et al. , 1988; also see Corbyn , 2011, for the African origins of humanity’s chemical research activities). Our metatheoretical calculus could try to capture this seemingly errant, if not outright vicious, (framing of this) heritage, and can attempt to answer questions as broad as: how does this constellation of fields delimit itself into pseudoscience and science through time?

Genealogical parallels or analogies can also be drawn, such as between alchemy’s quest for turning base metals into gold or for immortality and artificial general intelligence’s quest for so-called superhuman-level artificial intelligence (cf. Monett et al. , 2020; Gobble , 2019). In such a light, we can invite introspection or dialogue on if AI in this form is a science, is virtuous, etc. The same goes for the relationships between eugenics, physiognomy, or other ancestral $\theta$-, $\boldsymbol{\Theta }$-, and $\mathbb {T}$-theories to modern computational sciences generally, and psychology and genetics specifically (see Fig. 3 herein, as well as Birhane and Guest , 2021; Spanton and Guest , 2022; Yakushko , 2019; TallBear , 2013; Erscoi et al. , 2023; Scheuerman et al. , 2021).

Formalising such aspects can aid in answering for ourselves: what effect does the uncomfortable, oppressive heritage of AI, statistics, and psychology have on their modern theorising? What kinds of steps need to be taken to curb, stop, or reverse these harms (e.g. see right side of Fig. 3)? What is it about the genealogical links between the theory of evolution and eugenics that causes the latter to keep reappearing (see Rose , 2009)? Is, for example, changing the name of the journal (founded by Karl Pearson^5^) from *Annals of Eugenics* to *Annals of Human Genetics* (Stigler, 2010) enough? Or contrariwise, is it aiding in hiding history, the descent of theory and of research institutions, exactly where and when it should be documented, discussed, and explicitly condemned (e.g. Weigmann , 2001; Weindling et al. , 2021; Burke and Castaneda , 2021)? Are fields that use statistical and/or AI methods uncritically (e.g. Mukharji , 2015; Taylor et al. , 2021) not only obfuscating their genealogy, but also maintaining within their essence the potential to re-enact past and present documented harms?

## Where Do We Go from Here?

Could the categories I routinely use to conduct my research represent a problem that I am not aware of and that I should therefore identify as such so that I can address it?

Audrey Alejandro, 2021

Thinking about creating and explicitly stating how, or even if, we adjudicate over the virtuousness of our theories requires two basic metatheoretical components: (1) the hard work of both teasing theories apart from their contexts, understanding their cultural environs, and of dissecting them into vicious and virtuous; (2) identifying which vices and virtues we are interested in taking note of, or further minimising and maximising. Working through, and iterating over, these interrelated steps results in having and refining metatheoretical calculi.

If one only cares about, for example, goodness-of-fit to data, that is like saying one only counts the number of, e.g. bricks constituting their home and not any other features. This means a pile of bricks might be seen through a potentially flawed framing and metric as a superior house than an actual dwelling composed of other materials altogether or fewer bricks than the pile (cf. Forscher , 1963). The same goes for our theoretical housing in science. If we focus merely on a single metric, we impoverish ourselves, we leave our field(s) open to harm, and we risk the future of science itself. Because of this tendency, our focus on single traditional theoretical virtues (such as parsimony or goodness-of-fit, cf. Longino , 1996), I have forced a zooming out, wherein instead of concerning ourselves with individual such characteristics of theories we can look at whole ontologies first. Thus, science practitioners are allowed to answer the titular question by building up a series of virtues and vices as we see fit using our own formalisable metatheoretical calculus, i.e. an account of our adjudication over theories. The ontology of criteria, summarised in Table 1, contains the following: (a) metaphysical commitment, (b) discursive survival, (c) empirical interface, and (d) minimising harm. Theoretical scientists should show their theoretical work clearly, like empirical scientists should show their data and statistical work clearly. To be theoretical, in other words, is to demonstrate through metatheoretical calculi our adjudication over theories. If we wish to work on theory, then we should accessibly and transparently show our metatheory; whatever that is, I do not prescribe a specific meta-methodology. Just like if we work on data, as scientists, we should show our meta-to-the-data work, like the relevant statistical analyses or theoretical framing that house our experiments and hypotheses (viz. Guest and Martin , 2021).

Through our bespoke lens, as promoted herein, practitioners are invited to build up their formal metatheoretical calculus, including by proposing their own additional criteria and by consistently and coherently iterating over them as theories change. Therefore, we can try to avoid the precarity of basing our judgements of what is and is not a good theory on the shifting sands of hype (scientific objects often go in and out of fashion for non-scientific reasons), of human memory error (we may forget why we chose a specific theory to work under), of venture or other forms of capitalism (scientific so-called advances are fuelled often by their relationship to hegemonic forces and not through theory-driven rationale), etc. Ultimately, if formal modelling is useful for our theories, it should be just as useful for our theories about our theories. Such metatheoretical calculi can hopefully allow us to answer for ourselves: what makes a good theory, and how do we make a theory good?

## Data Availability

Not applicable

## Footnotes

1 The author would like to thank Yue Chen for highlighting the potential for a misunderstanding about proceduralisation. I do not condone nor propose automation. Also, see Rich et al. (2021) and Okasha (2011) for why this is impossible anyway.

2 This is not to purposefully exclude, for example, causality-free theory from being theory, but to explicate to which kinds of theory I apply the virtues herein. If subjecting, e.g. causality-free theory, perhaps as found in mathematics, to these virtues is a fruitful lens, then the reader is welcome to do so.

3 Icons by *a* & *b*) iconixar, *c*) amonrat rungreangfangsai, and *d*) BomSymbols.

4 Here, this term is used in the (informal) sense that a theory is so expressive that it can account for any computational process (see Arbib 1987). Thus, the theory is so expressively powerful that it can likely account for anything.

5 Pearson is described often as a father of modern statistics along with Ronald Fisher and Francis Galton, all of whom were pioneer eugenicists (Clayton, 2020).
